# Constructive approach for synthesis of a functional IgG using a reconstituted cell-free protein synthesis system

**DOI:** 10.1038/s41598-018-36691-8

**Published:** 2019-01-24

**Authors:** Satoshi Murakami, Rena Matsumoto, Takashi Kanamori

**Affiliations:** GeneFrontier Corporation, Kashiwa, Chiba 277-0882, Japan

## Abstract

IgG is an indispensable biological experimental tool as well as a widely-used therapeutic protein. However, cell culture-based expression of monoclonal IgG is costly and time-consuming, making this process difficult to use for high-throughput screening in early-stage evaluation of biologics. With the goal of establishing a fast, simple, and robust high-throughput expression system for IgG, we implemented the synthesis of functional aglycosylated IgG by constructive approach based on a reconstituted prokaryotic cell-free protein synthesis system (PURE system). Optimization of the PURE system revealed that the following factors and reaction conditions were needed for IgG synthesis: (1) inclusion of the disulfide bond isomerase DsbC, (2) adjustment of the GSH/GSSG ratio, (3) inclusion of the molecular chaperone DnaK and its cofactors, and (4) use of an extended incubation time. Synthesis temperature and template DNA ratio (light chain-/heavy chain-encoding) also had been optimized for each IgG. Under optimal conditions, peak production of the anti-HER2 antibody trastuzumab reached 124 µg/mL. Furthermore, the active forms of other IgGs, including IgG1, IgG2, and IgG4 subclasses, also were synthesized. These results provide basic information for the development of novel high-throughput expression and functional screening systems for IgG, as well as useful information for understanding the IgG synthesis process.

## Introduction

Immunoglobulin G (IgG) is an indispensable experimental tool in current basic biology research, where this molecule is used for its specific binding ability and high affinity for antigens. Concomitantly, monoclonal IgGs continue to attract attention as a dominant therapeutic protein for the diagnosis and treatment of a variety of diseases in the global biopharmaceutical market^1^. IgG is a large, complex, Y-shaped heterotetrameric protein consisting of two identical light chains (LCs) and two identical heavy chains (HCs) connected by disulfide bonds^2^. Each chain is organized in multiple Ig domains, which can be categorized in turn into variable (VL, VH) and constant (CL, CH1, CH2, CH3) domains. In mammalian B cells or plasma cells, both LC and HC are co-translationally translocated into the endoplasmic reticulum (ER) and form a whole IgG via a multiple-step folding and subunit assembly process^3^. The complicated folding of IgG and associated quality control processes are assisted by ER proteins such as the ER chaperone BiP, peptidyl-prolyl *cis*-*trans* isomerase (PPIase), protein disulfide isomerase (PDI), and their cofactors^3^.

Due to the complexity of IgG synthesis, various well-established mammalian cell culture systems (such as murine hybridoma, Chinese hamster ovary (CHO) cells, and HEK293 cells) have been employed for the development and production of monoclonal IgG, from the laboratory scale to the industrial scale. Cell culture-based systems, however, include multiple steps, and can require intervals ranging from several days to several months to obtain cells transiently or stably expressing recombinant monoclonal IgG, rendering these processes costly and time-consuming, especially for high-throughput expression. These challenges represent bottlenecks in the early stages of development of therapeutic antibodies. One solution has been the high-throughput expression and functional screening of monoclonal antibodies using cell-free protein synthesis platforms. Antibody fragments such as single-chain variable fragment (scFv) and fragment antigen-binding (Fab) can be synthesized with cell lysate-based cell-free systems derived from wheat germ^4^, insect cells^5,6^, or *Escherichia coli*^7–11^. In the past decade, several reports have described the synthesis of whole IgG using cell lysate-based cell-free systems derived from *E*. *coli*^11–13^, plant cell culture^14^, or CHO cells^15,16^. Moreover, Groff *et al*. reported the synthesis of aglycosylated IgG at gram-per-liter scale by using an improved *E*. *coli* S30 extract-based system^13^.

The cell lysate-based cell-free system is relatively cost- and time-effective and scalable for the purpose of expression of monoclonal IgG compared to mammalian cell culture. However, because unpurified cell lysate is used for the system, the reaction mixture contains cell-derived components with concentrations varying by lysate preparation conditions and cellular source. The lysate includes not only substances related to protein synthesis but also organelles (e.g., ER reformed as microsomes^5,6,14,16^), interfering substances (e.g., nucleases, proteases, and other degradative and metabolic enzymes), and unintended protein synthesis-promoting factors (e.g., chaperones and unknown protein-folding and -stabilizing factors). This crude background impedes the establishment of more-robust, high-throughput expression and simpler functional screening systems for the following reasons: (1) It is difficult to identify substances that interfere with IgG synthesis for selective inhibition or removal from the mixture. (2) Since the crude background may interfere with the subsequent functional analyses such as binding kinetics and cell-based assays, a process for high-level purification of the synthesized product is essential. Therefore, identification of the minimal factors essential for cell-free synthesis of IgG is expected to permit the establishment of a simple and robust expression system, facilitating further improvement of the system while also potentially permitting functional screening of the products even in the absence of purification. Thus, a constructive “bottom-up” approach, depending on systematic assembly of the synthetic components (rather than generating a lysate by breaking down from the cell), is expected to be useful.

The PURE (Protein synthesis Using Recombinant Elements) system is a reconstituted cell-free protein synthesis system based on the protein synthesis machinery of *E*. *coli*^17^. Unlike the cell lysate-based system, the PURE system contains only purified factors involved in transcription, translation, and energy regeneration. In the PURE system, the number of cell-derived contaminants is greatly decreased; reagent composition and reaction conditions can be readily adjusted. Thus, the PURE system is expected to be useful for evaluating protein synthesis and subsequent folding reactions under conditions that exclude the influence of contaminants^18^, facilitating functional analysis of the synthesized protein even in the absence of purification.

**Figure 1:**
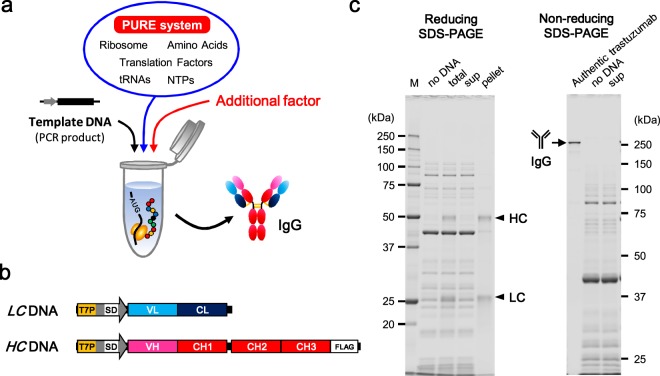
IgG synthesis with the standard PURE system. (**a**) Schematic of the concept of this study’s synthesis of IgG with the PURE system. (**b**) Template DNA design of trastuzumab used in this study. T7P, T7 promoter; SD, Shine-Dalgarno sequence. (**c**) Results of the synthesis of trastuzumab using the standard PURE system. After synthesis, the reaction mixture (total) was centrifuged and separated into supernatant (sup) and pellet. Samples were subjected to reducing and non-reducing SDS-PAGE with different gels (12.5% and 10% gel, respectively) and subsequently imaged by fluorescent staining. Authentic trastuzumab was loaded at 100 ng/lane. M, molecular weight marker.

Therefore, in the present study, we attempted the synthesis of whole IgG by the addition of minimal factors to the PURE system (Fig. 1a), permitting the definition of the minimal additional factors and the optimum reaction conditions for the synthesis of functional aglycosylated IgGs. Several IgGs, including IgG1, IgG2, and IgG4 subclasses, also were synthesized in a limited number of steps using the optimized PURE system; the binding activities of these products were confirmed even without purification. Our results provide valuable information regarding the IgG synthesis process, and a useful basis for further engineering of the IgG synthesis process. Therefore, our results indicate the applicability of the constructive approach, based on the PURE system, for establishing simple and robust high-throughput expression and functional screening of antibodies.

## Results

### Synthesis of IgG using the standard PURE system

All experiments in this paper used the commercially-available PURE*frex*^®^ *2*.*0* kit as the standard PURE system. The well-known anti-HER2 antibody trastuzumab^19^ was selected as a model IgG for the optimization of synthesis conditions. Since the PURE system does not contain nuclease activity, linear (e.g., PCR product) or circular DNA can be used as the expression template. Therefore, LC- and HC-encoding template DNAs were constructed in a T7 promoter-driven PCR product format (Fig. 1b). As a first step, we tested whether IgG can be synthesized with the standard PURE system with only basic protein translation function. *LC* and *HC* template DNAs were simultaneously added to a total of 10 nM of the mixed PCR products (molar ratio of *LC*:*HC* = 1:1) to the PURE system and the reaction mixture was incubated at 37 °C for 16 hours. After centrifugation, the reaction mixture was subjected to reducing and non-reducing SDS-PAGE following by detection and quantitation in the gel by staining with fluorescent dye (Fig. 1c). As shown in reducing SDS-PAGE, both LC (23.6 kDa) and HC (50.6 kDa) were synthesized (to 166 µg/mL and 116 µg/mL, respectively) using the standard PURE system without optimization. However, all of the synthesized LC and HC were pelleted by centrifugation (lane “pellet”); notably, whole IgG (148.3 kDa) was not observed on non-reducing SDS-PAGE (lane “sup”). This result indicated that the synthesized LC and HC needed to be solubilized to permit assembly of whole IgG. Therefore, the improvement of IgG synthesis by constructive approach based on the PURE system was attempted, as follows.

### Improvement of solubility and formation of disulfide bonds

**Figure 2:**
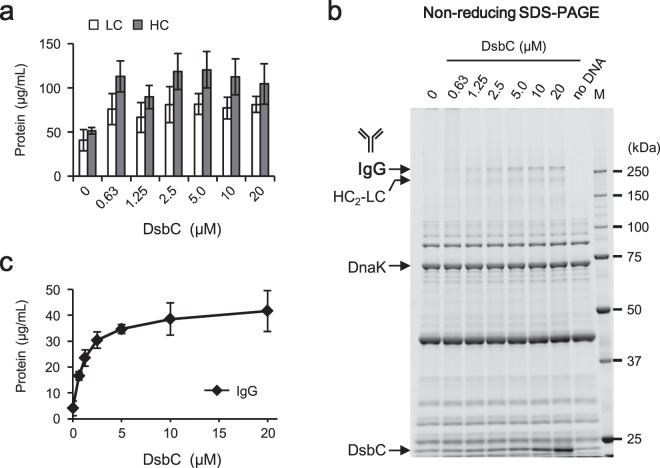
Optimization of DsbC concentration. The reactions were performed using the PURE system supplemented with 0 to 20 µM DsbC, 3 mM GSSG, 1× DnaK mix, and 10 nM mixed template DNA (molar ratio of *LC*:*HC* = 1:1). Incubation was performed at 37 °C for 16 hours. After centrifugation, the supernatant was subjected to quantitative analysis. Specifically, the total soluble LC and HC (**a**) and the whole IgG (**b**,**c**) in the same sample were quantitated with reducing and non-reducing SDS-PAGE. All data represent the mean and standard deviation of three independent experiments. M, molecular weight marker.

In separate work (Supplementary Fig. S1), we showed that Fab and scFv derived from trastuzumab could be synthesized in soluble and active forms by adding a molecular chaperone DnaK and its cofactors (DnaK mix) and a disulfide bond isomerase DsbC to the reaction mixture. Therefore, enhancement of IgG synthesis with the PURE system was attempted here using the same approach. First, DnaK mix was added to the PURE system to improve the solubility of LC and HC. DnaK is a cytoplasmic molecular chaperone HSP70 from *E*. *coli*^20,21^ and the 1× DnaK mix consists of 5 μM DnaK and its cofactors DnaJ and GrpE at 1 μM each. Both the synthesized LC and HC were partially solubilized by addition of this molecular chaperone (“0 μM DsbC” in Fig. 2a). The amounts of soluble LC and HC were 41 ± 12 µg/mL and 51 ± 4 µg/mL (mean ± SD), respectively. However, whole IgG was not observed with non-reducing SDS-PAGE (“0 μM DsbC” in Fig. 2b,c). This result indicated that another challenge for IgG synthesis with the PURE system was how to promote the formation of intermolecular disulfide bonds between LCs and HCs.

Next, DsbC, which promotes correct disulfide bond formation in the *E*. *coli* periplasm^22–24^, was added at various concentrations to the PURE system. Specifically, the synthesis reactions were performed with the PURE system supplemented with 0 to 20 μM DsbC in the presence of 1× DnaK mix and 3 mM GSSG as a source of oxidizing activity. Synthesized LC and HC were solubilized to higher levels in the presence of DsbC (Fig. 2a). The amounts of soluble LC and HC obtained with 0.63 μM DsbC were 76 ± 17 µg/mL and 113 ± 18 µg/mL, respectively. Whole IgG was not observed without DsbC, but was detected at increasing concentrations as the DsbC concentration rose (Fig. 2b,c). The HC_2_-LC trimer also was observed as a band smaller than that of whole IgG. IgG was fully synthesized (at 35 ± 2 µg/mL) in the presence of at least 5 µM DsbC. This result suggested that DsbC plays a critical role in the formation of intermolecular disulfide bonds as well as in the solubilization of LC and HC.

### Optimization of the redox state

**Figure 3:**
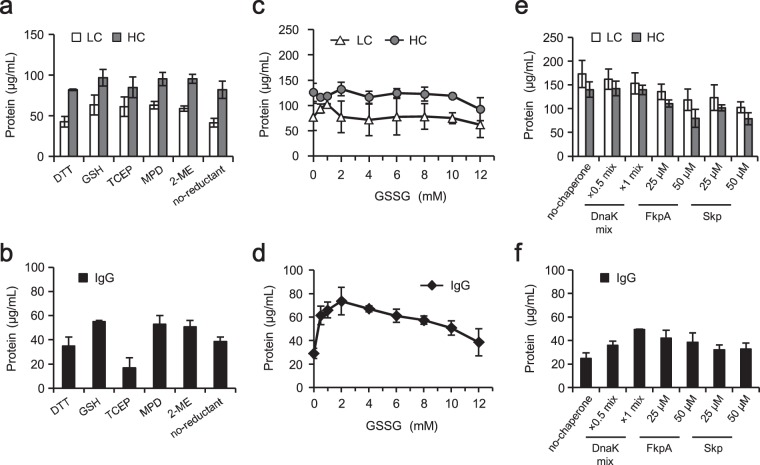
Optimization of redox state and chaperones. (**a**,**b**) The effect of reducing agents on IgG synthesis. The reaction was performed with the PURE system containing one of the indicated reducing agents (at 2 mM), 3 mM GSSG, 5 μM DsbC, 1× DnaK mix, and 10 nM mixed template DNA (molar ratio of *LC*:*HC* = 1:1). Incubation was performed at 37 °C for 16 hours. TCEP, tris (2-carboxyethyl) phosphine; MPD, 3-mercapto-1,2-propanediol; 2-ME, 2-mercaptoethanol. (**c**,**d**) Optimization of GSSG concentration. The reaction was performed with the PURE system containing 2 mM GSH instead of DTT, 0 to 12 mM GSSG, 5 µM DsbC, 1× DnaK mix, and 10 nM mixed template DNA (molar ratio of *LC*:*HC* = 1:1). Incubation was performed at 37 °C for 16 hours. (**e**,**f**) The effect of molecular chaperones and chaperone-like proteins on IgG synthesis. The reaction was performed with the PURE system containing 2 mM GSH instead of DTT, 3 mM GSSG, 5 µM DsbC, the indicated chaperones, and 10 nM mixed template DNA (molar ratio of *LC*:*HC* = 1:1). Incubation was performed at 37 °C for 16 hours. After centrifugation, the supernatant was subjected to quantitative analysis. The total soluble LC and HC (**a**,**c**,**e**) and the whole IgG (**b**,**d**,**f**) in the same sample were quantitated with reducing and non-reducing SDS-PAGE. All quantitated data represent the mean and standard deviation of three independent experiments.

The PURE system usually contains 1–3 mM DTT as a reducing agent^17^; the redox state of the reaction can be controlled by altering the concentration and the ratio of reducing and oxidizing agents. As a next step, the effect of the reducing agent on IgG synthesis with the PURE system was examined (Fig. 3a,b). Synthesis reactions were performed in the presence of 2 mM of various reducing agents in combination with 3 mM GSSG, 5 μM DsbC, and 1× DnaK mix. Different reducing agents did not yield apparent effects on the amounts of synthesized LC and HC (Fig. 3a). However, the amount of IgG generated varied depending on the reducing agent employed (Fig. 3b). When DTT or Tris (2-carboxyethyl) phosphine (TCEP) was added to the reaction mixture, the amount of IgG generated was about the same or lower than that obtained without reducing agent. GSH gave a relatively larger amount of IgG among the tested reducing agents.

The concentration of GSSG as an oxidizing agent then was optimized (Fig. 3c,d). Synthesis reactions were performed with the PURE system containing 0 to 12 mM GSSG in the presence of 2 mM GSH, 5 μM DsbC, and 1× DnaK mix. There was no effect of the concentration of GSSG on the amounts of synthesized LC and HC (Fig. 3c), while the highest amount of IgG was observed at GSSG concentrations between 0.5 and 4.0 mM (Fig. 3d). Based on these results, GSH and GSSG were used at a molar ratio of 2/3 mM in the susbsequent experiments. These results indicated that the type of reducing agent and the ratio of reducing and oxidizing agent were important for IgG formation.

### Effect of chaperone and chaperone-like proteins

Chaperone-like proteins FkpA and Skp have been reported to have significant effects on the synthesis of functional antibodies when scFv or Fab was co-expressed with FkpA or Skp^25–29^. FkpA, a periplasmic PPIase from *E*. *coli*, has a chaperone-like function independent of the PPIase activity^24,30^. Skp, a periplasmic protein from *E*. *coli*, shows chaperone-like activity for β-barrel proteins^24^. The effect of chaperone and chaperone-like proteins on IgG synthesis was examined in the presence of 2 mM GSH, 3 mM GSSG, and 5 μM DsbC (Fig. 3e,f). The amounts of soluble LC and HC were almost the same when the reactions were performed in the presence (“DnaK mix”) and absence (“no-chaperone”) of the DnaK mix and were slightly decreased in the “FkpA” and “Skp” reactions (Fig. 3e). Since both LC and HC were completely solubilized in the presence of 5 μM DsbC, further solubilization by the addition of chaperone was not observed. On the other hand, the amount of IgG varied depending on the added chaperones (Fig. 3f). DnaK mix gave the highest amount of the IgG with 1× mix. Both FkpA and Skp also had effects on IgG formation, although the effect was smaller than that obtained with 1× DnaK mix. This result suggested that DnaK was the most suitable chaperone for IgG formation among those tested. Furthermore, using several IgGs, including trastuzumab, we tested whether the DnaK mix and FkpA exhibited an additive effect on IgG formation. In the presence of both 1× DnaK mix and FkpA, however, the yield of trastuzumab whole IgG was decreased compared to that obtained with the 1× DnaK mix alone, and whole IgGs of the other molecules were not generated with the combination of 1× DnaK mix and FkpA (Supplementary Fig. S2).

### Optimization of the synthesis reaction condition

**Figure 4:**
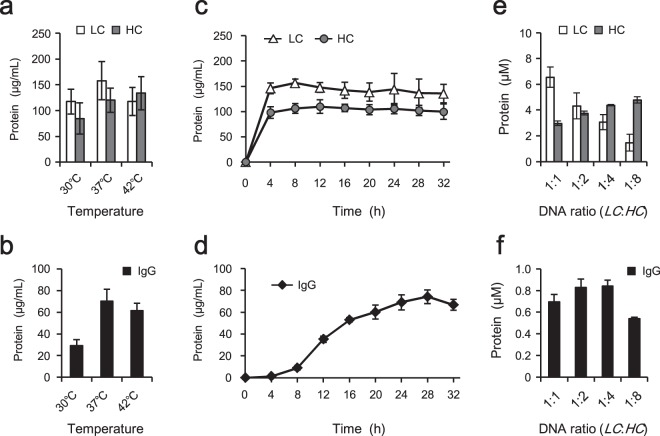
Optimization of the synthesis reaction conditions and DNA ratio. (**a,b**) Optimization of the incubation temperature. The reactions were performed with the PURE system containing 2 mM GSH instead of DTT, 3 mM GSSG, 5 μM DsbC, 1× DnaK mix, and 10 nM mixed template DNA (molar ratio of *LC*:*HC* = 1:1). Incubation was performed at 30, 37, or 42 °C for 16 hours. (**c**,**d**) Optimization of the incubation time. The above reaction mixture was incubated at 37 °C for 0 to 32 hours. (**e**,**f**) Optimization of the template DNA ratio. The above reaction mixture containing the indicated molar ratio of *LC* DNA and *HC* DNA (total 10 nM of mixed template DNA) was incubated at 37 °C for 28 hours. After centrifugation, the supernatant was subjected to quantitative analysis. The total soluble LC and HC (**a**,**c,e**) and the whole IgG (**b**,**d,f**) in the same sample were quantitated with reducing and non-reducing SDS-PAGE. All quantitated data represent the mean and standard deviation of three independent experiments.

The optimal synthesis temperature of the PURE system is usually 37 °C, but for synthesis of aggregation-prone proteins, lower temperature (e.g., 30 °C) can prevent aggregation. Therefore, the optimal synthesis temperature of trastuzumab was examined by performing the reactions at 30, 37, or 42 °C for 16 hours (Fig. 4a,b). The amount of IgG was highest at 37 °C, although product also was synthesized at 30 °C and even at 42 °C.

To examine the effect of incubation time, trastuzumab was synthesized for up to 32 hours at 37 °C (Fig. 4c,d). Protein synthesis in the PURE system usually plateaus after 4 hours at 37 °C. Likewise, the synthesis of LC and HC reached a plateau at 4 hours (Fig. 4c). However, no IgG was observed at that time (Fig. 4d). IgG was observed starting at 8 hours, with the level of the multimer gradually increasing through 28 hours. The highest amount of IgG was observed at around 28 hours, when the yield reached 74 ± 6 µg/mL. This result indicated that the formation of intermolecular disulfide bonds between the LCs and HCs required extended incubation under these reaction conditions.

### Optimization of LC:HC DNA ratio

In simultaneous synthesis of multiple components of a protein using the PURE system, the ratio of the synthesized components can be controlled by changing the ratio of the template DNAs^31^. To maximize the yield of whole IgG, therefore, the molar ratio of *LC* DNA and *HC* DNA was optimized while holding the total template concentration at 10 nM (Fig. 4e,f). The amounts of synthesized LC and HC correlated with the ratio of the template DNAs (Fig. 4e). When the molar ratio of *LC* DNA and *HC* DNA was 1:2 to 1:4, the molar concentrations of synthesized LC and HC were approximately 1:1; the amount of IgG also was highest under these conditions (Fig. 4f).

### Summary of the optimization of IgG synthesis

**Figure 5:**
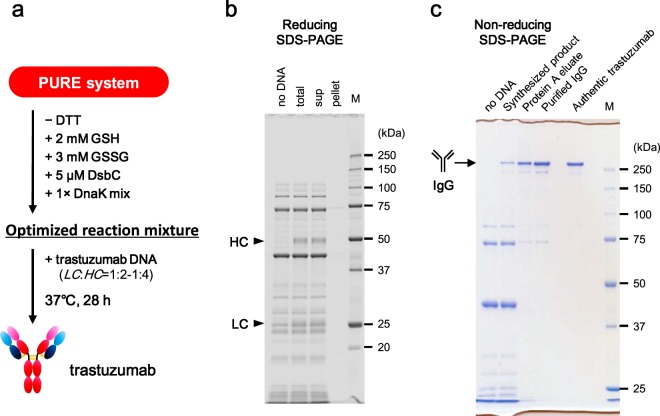
Synthesis and purification of trastuzumab. (**a**) An overview of the optimized synthesis conditions for trastuzumab. (**b**,**c**) Synthesis of trastuzumab using the optimized PURE system and purification of the synthesized products. After synthesis, the reaction mixture (total) was centrifuged and separated into supernatant (sup) and pellet. The samples were subjected to reducing SDS-PAGE (12.5% gel) and subsequent fluorescent staining (**b**). Synthesized product was purified with Protein A resin (Protein A eluate) and subsequent gel filtration (Purified IgG). The samples were subjected to non-reducing SDS-PAGE (10% gel) and subsequent Coomassie Brilliant Blue (CBB) staining (**c**). Authentic trastuzumab was loaded at 500 ng/lane. M, molecular weight marker.

A summary of the optimization of IgG synthesis with the PURE system is shown in Fig. 5a. In the absence of DTT, the PURE system supplemented with 2 mM GSH, 3 mM GSSG, 5 μM DsbC, and 1× DnaK mix was a suitable reaction mixture for the synthesis of the trastuzumab IgG. The optimal molar ratio of *LC* DNA and *HC* DNA for trastuzumab was 1:2 to 1:4 when the total template concentration was set at 10 nM. The optimal incubation condition was 37 °C for 28 hours. Both synthesized LC and HC were solubilized completely under the optimized conditions (Fig. 5b), and whole IgG was observed in the same position as authentic trastuzumab (“Synthesized product” in Fig. 5c). The amounts of synthesized LC and HC in Fig. 5b were 84 µg/mL and 132 µg/mL, respectively. The yield of the whole trastuzumab IgG reached 124 ± 9 µg/mL (Table 1). IgG formation efficiency (the proportion of LC and HC which formed IgG out of the total synthesized LC and HC molecules; see Formula (1) was about 45% under these conditions (Table 1).

**Table 1:** Summary of the synthesis of several IgGs with the optimized PURE system.

Name	Subclass	Antigen	MW (kDa)	Optimal reaction condition		Yield* (µg/mL)	IgG formation efficiency** (% (mol/mol))	EC_50_*** (nM)
				Temperature	DNA ratio (*LC*:*HC*)			
trastuzumab	IgG1κ	HER2	148.3	37 °C	1:2–1:4	124 ± 9	45%	0.16
adalimumab	IgG1κ	TNF-α	147.4	37 °C	1:2–1:4	46 ± 6	14%	0.1
cetuximab	IgG1κ	EGFR	148.3	30 °C	2:1	49 ± 6	30%	0.02
panitumumab	IgG2κ	EGFR	144.9	37 °C	2:1	33 ± 3	12%	0.036
nivolumab	IgG4κ	PD-1	144.2	30 °C	1:2	73 ± 2	26%	0.05

*Values are expressed as mean ± SD (n = 3).

**The proportion of LC and HC which formed IgG out of total LC and HC molecules synthesized under the optimal condition.

***50% effective concentration of target binding activity in ELISA.

### Evaluation of the activity of synthesized trastuzumab

Trastuzumab synthesized with the optimized PURE system was purified by Protein A resin and subsequent gel filtration (Fig. 5c). As a result, 131 µg of trastuzumab was obtained from 1 mL of the optimized reaction mixture, and 68 µg of purified IgG was obtained using a standard method for IgG purification. Purification yield was 75.4% at the Protein A eluate step and 51.7% at the subsequent gel filtration step.

**Figure 6:**
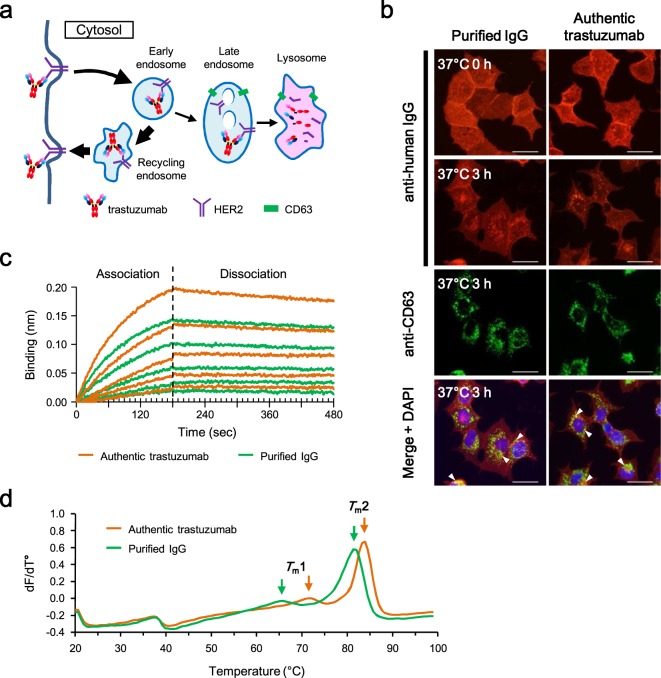
Evaluation of the activity of purified trastuzumab. (**a**) A model of trastuzumab uptake and intracellular trafficking in HER2-expressing BT-474 cells. (**b**) Internalization analysis of purified trastuzumab. BT-474 cells were surface-labeled at 4 °C for 60 min with 10 nM purified IgG or authentic trastuzumab. Cells were washed and incubated at 37 °C for 0 or 3 hours, and then fixed and processed by dual-label indirect immunofluorescence microscopy. CD63 (Lamp3) is a marker of late endosomes and lysosomes. White arrowheads indicate partial co-localization of trastuzumab with CD63. Bar indicates 20 μm. (**c**) Binding kinetics of purified trastuzumab. Binding kinetics was measured by biolayer interferometry on an Octet RED96 system. Purified IgG and authentic trastuzumab were loaded onto Anti-Human IgG Fc Capture biosensor, and affinities of the antibodies were measured using serial dilutions of recombinant HER2 protein. (**d**) Thermal stability of purified trastuzumab. Purified IgG and authentic trastuzumab were subjected to thermofluor assay using ProteoStat Thermal Shift Stability Assay kit and LightCycler 480 system. *T*_m_1 and *T*_m_2 represent the aggregation temperature.

The activity of purified trastuzumab was evaluated. HER2, a transmembrane tyrosine kinase, is a binding target of trastuzumab and is overexpressed on the plasma membrane in numerous human breast cancer cells. In the breast cancer cell line BT-474, the surface-displayed HER2 bound by trastuzumab is internalized into the cells by endocytosis (Fig. 6a). It has been reported that a large portion of these HER2 receptors are returned to the cell-surface by the recycling endosome, with only a small fraction of the receptors entering into the lysosomal degradative pathway^32^. The activity of PURE-synthesized trastuzumab was tested by assessing internalization into BT-474 cells (Fig. 6b). Surface-labeled BT-474 cells with purified PURE-synthesized trastuzumab were incubated at 37 °C for 0 or 3 hours. Cells were then fixed and processed by dual-label indirect immunofluorescence microscopy. The visualized IgG was only observed on the cell surface before incubation (0 h). On the other hand, perinuclear dot stains following internalization were observed in addition to cell-surface staining after incubation (3 h). The dot stains partially co-stained with the late endosome and lysosome marker CD63 (Lamp3). Thus, the purified IgG showed internalization similar to that seen with authentic trastuzumab.

The binding affinity of purified IgG was measured by using biolayer interferometry (Fig. 6c). The purified IgG was loaded onto the Anti-Human IgG Fc Capture biosensor and affinity of the biosensor was measured against a serial dilution of recombinant HER2 protein. The dissociation constant (*K*_D_) of purified IgG was 4.24E-10 M, and the on-rate (*k*_on_) and the off-rate (*k*_off_) were 6.38E + 05 M^−1^s^−1^ and 2.70E-04 s^−1^, respectively. These values were similar to those obtained in the same assay using authentic trastuzumab (*K*_D_: 4.03E-10 M, *k*_on_: 6.29E + 05 M^−1^s^−1^, *k*_off_: 2.53E-04 s^−1^).

The thermal stability of purified IgG was measured by thermofluor assay using a fluorescent dye that binds to hydrophobic patches exposed as the protein unfolds (Fig. 6d). The aggregation temperature measured by this assay matches closely with *T*_m_ as measured by differential scanning calorimetry (DSC), and *T*_m_ of trastuzumab measured in this assay was reported as 85.1 °C^33^. The aggregation temperature of purified IgG shifted to a little lower side compared with that of authentic trastuzumab (purified IgG, *T*_m_1: 65 °C, *T*_m_2: 81 °C; authentic trastuzumab, *T*_m_1: 71 °C, *T*_m_2: 84 °C). This difference may be due to the lack of post-translational modification. PURE-synthesized IgG has no sugar chains and the absence of sugar chains is known to lower the thermal stability of IgG^34,35^.

These results demonstrated that trastuzumab synthesized using the optimized PURE system had activity similar to that of authentic trastuzumab produced with CHO cells although it is a little less thermostable.

### Synthesis of other IgGs with the optimized PURE system

**Figure 7:**
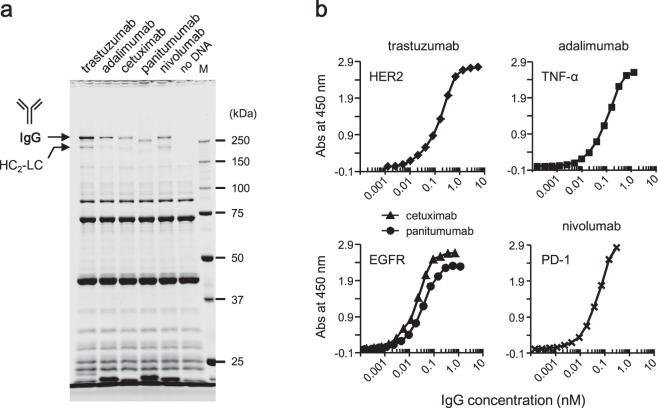
Synthesis of various IgGs. (**a**) Results of the synthesis of several IgGs using the optimized PURE system. The synthesis reactions were carried out with the PURE system containing 2 mM GSH instead of DTT, 3 mM GSSG, 5 μM DsbC, DnaK mix, and 10 nM mixed template DNA. Incubation was performed for 28 hours. Synthesis temperature and template DNA ratio (molar ratio of *LC*:*HC*) were optimized for the individual IgGs as shown in Table 1. After centrifugation, the supernatant was subjected to non-reducing SDS-PAGE (10% gel). M, molecular weight marker. (**b**) Analysis of binding of synthesized IgGs to each antigen. The reaction mixtures including the synthesized IgGs were serially diluted and applied to ELISA without purification.

In addition to trastuzumab, other IgGs, including IgG1, IgG2, and IgG4 subclasses, were synthesized using the optimized PURE system. All tested IgGs were synthesized with the optimized reaction mixture described in Fig. 5a, exhibiting yields in the range of 33 ± 3 to 73 ± 2 µg/mL under the product-specific optimal conditions (Fig. 7a and Table 1; also see next paragraph). The binding affinity of these synthesized IgGs could be evaluated even without purification. Reaction mixtures containing the synthesized IgGs were serially diluted and tested by ELISA (Fig. 7b). Notably, the synthesized IgGs exhibited high binding affinity for their respective native target antigens, exhibiting affinities (EC_50_) in the sub-nanomolar range (Table 1). Cross-reactivity to other antigens was not observed for any of the tested cases (Supplementary Fig. S3).

The best conditions for the synthesis reactions differed among the tested IgGs. Testing of the synthesis temperature revealed that the amounts of IgG of adalimumab (IgG1) and panitumumab (IgG2) were the highest at 37 °C; both could be synthesized at 30 °C but not at 42 °C. (Table 1 and Supplementary Fig. S4). Cetuximab (IgG1) and nivolumab (IgG4) could be synthesized at 30 °C, but not at above 37 °C. The optimal template DNA ratio also differed for the different products (Table 1 and Supplementary Fig. S5). Adalimumab and nivolumab required more *HC* DNA to maximize the IgG yield, while cetuximab and panitumumab required more *LC* DNA than *HC* DNA. The IgG formation efficiency of the IgGs ranged from 12 to 30% under the respective optimal conditions (Table 1). Remarkably, despite having completely identical amino acid sequences outside of the variable regions (VL and VH), yields and optimal conditions differed between IgG1 members trastuzumab, adalimumab, and cetuximab. Therefore, the difference in optimal conditions between individual IgGs reflected differences in the complementarity-determining region (CDR) sequences and structures.

These results suggest that it is possible to synthesize various functional IgGs by using the optimized PURE system, although the synthesis temperature and the template DNA ratio will need to be optimized for individual IgGs for the best yields.

## Discussion

In this study, we implemented the synthesis of a functional aglycosylated IgG by a constructive approach based on a reconstituted prokaryotic cell-free protein synthesis system (PURE system) with the goal of establishing a fast, simple, and robust high-throughput expression system for IgG. As a result, the following minimal additional factors and reaction conditions for the synthesis of functional aglycosylated IgG using the PURE system were determined: (1) inclusion of disulfide bond isomerase (5 µM DsbC), (2) use of GSH instead of DTT as a reducing agent and adjustment of the GSH/GSSG ratio to 2/3 mM, (3) inclusion of a molecular chaperone and its cofactors (5 µM DnaK, 1 µM DnaJ, and 1 µM GrpE), and (4) use of an extended incubation interval (28 hour). The results also suggested that the synthesis temperature and the template DNA ratio (molar ratio of *LC*:*HC*) should be optimized for individual IgGs to ensure the best yields. The PURE system comprises only those purified factors necessary for transcription, translation, and energy regeneration^17^. Therefore, to the best of our knowledge, the PURE system specified in this study represents the minimum configuration for IgG synthesis. These results provide basic information for understanding the IgG folding/assembling process and are expected to facilitate further improvement of the IgG production process.

In terms of the folding process, several aspects of the composition and reaction conditions of the PURE system needed to be optimized to ensure the most efficient synthesis of IgG. The addition of the 1× DnaK mix exerted a beneficial effect, improving the solubility of synthesized LC and HC and enhancing IgG formation. Frey *et al*. and Groff *et al*. both reported that BiP, which contributes to HC stabilization during the folding of IgG in the mammalian ER, had a little or no effect on IgG synthesis using a *E*. *coli* S30 extract-based cell-free system^12,13^. Those two papers hypothesized that the addition of another HSP70 (like BiP) had little effect because the expression system already included abundant endogenous DnaK and cofactors. Indeed, DnaK is one of the most abundant cytoplasmic molecular chaperones in prokaryotes; this protein is expressed constitutively in *E*. *coli* and is present at approximately 10,000 copies per cell under non-stressed condition^20^. Moreover, Bonomo *et al*. synthesized several Ig domain-containing proteins in the presence of BiP or DnaK with the PURE system; those authors reported that these two chaperones provided similar folding assistance^36^. Our results clearly indicated that DnaK and its cofactors are useful for IgG synthesis, which apparently serve in place of the eukaryotic BiP.

DsbC contributes to oxidative folding of proteins by promoting disulfide bond formation via thiol-disulfide exchange reaction^22–24^. The importance of DsbC in IgG synthesis and its interchangeability with eukaryotic PDI has been reported in cell lysate-based cell-free systems derived from *E*. *coli*^12,13^ or CHO cells^15^. Our results showed that addition of DsbC has a critical effect on cell-free synthesis of IgG, as reported in previous studies. DsbC also has a chaperone-like activity independent of isomerase activity^37^, which may further contribute to solubilization of synthesized LC and HC. In mammalian cells, it is known that IgG folding is assisted by several ER proteins such as BiP, PDI, PPIase and their cofactors^3^. Surprisingly, our results showed that the addition of DnaK mix and DsbC significantly improved the folding of IgG in the PURE system, which means that those molecules can work as some ER protein substitutes.

In IgG synthesis with the CHO cell lysate-based cell-free system, a GSH/GSSG ratio of 0.25/2 mM was indicated as the optimal condition^15^. Similarly, our results indicated that the relatively oxidative conditions, with GSH/GSSG ratios of 2/0.5 to 2/4 mM, were required for efficient IgG formation. On the other hand, we found that the type of reducing agent affected the formation of IgG, although the type of reducing agent did not affect the amounts of LC and HC that were synthesized. DTT and TCEP gave lower IgG yields than other tested reducing agents (Fig. 3b). The equilibrium constant $({K}_{{\rm{eq}}}^{{\rm{Obs}}})$ between DTT and GSSG at pH 7.0 is about 200 M^38,39^, while the value between GSH and GSSG or 2-ME and GSSG is 1 or 1.2 M^40^, respectively. Thus, oxidation of DTT by GSSG is almost irreversible due to imbalanced equilibrium under the condition described here. TCEP, a water-soluble trialkylphosphine, is an irreversible strong reducing agent^41^. The DTT and TCEP results suggest that maintaining the proper thiol-disulfide equilibrium state between reducing and oxidizing agents in the presence of DsbC is necessary for IgG formation.

The IgG formation efficiency of the tested IgGs was far from 100% (Table 1). The first cause of this result is the imbalance of the amount of the synthesized products of HC and LC molecules, presumably due to unstable CDR sequences. For example, in trastuzumab which has the highest IgG formation efficiency among IgG1 members in this experiment, the synthesized LC and HC molecules were approximately 1:1 at the maximum yield of IgG (Fig. 4e,f). However, in adalimumab and cetuximab, the synthesized LC and HC molecules were not 1:1 at the maximum yield of IgG (Supplementary Fig. S5). Since the presence of excess HC or LC molecules reduces IgG formation efficiency, it will be necessary to optimize the ratio and to stabilize the HC or LC molecules by optimizing the CDR sequences. As a second, since control of intermolecular disulfide bonding is very difficult *in vitro*, undesirable byproducts such as HC and LC monomer, dimer, HC_2_-LC trimer and nonspecific disulfide-bonded polymer are simultaneously formed with whole IgG. Moreover, the assembly of LCs and HCs into the heterotetramer required extended incubation (28 hours). In order to improve IgG formation efficiency and accelerate IgG formation more, addition of other factors to the optimized PURE system would be needed. Feige *et al*. have summarized the folding mechanism of IgGs, categorizing the folding of each Ig domain into three categories based on the folding process^3^. In all categories, prolyl *cis*-*trans* isomerization is commonly the rate-limiting step. Indeed, several *in vitro* studies have shown that the folding of the Ig domain is accelerated in the presence of PPIase^42–44^. However, the simultaneous addition of 1× DnaK mix and *E*. *coli* PPIase FkpA to the PURE system did not accelerate IgG formation in our experiments, instead lowering the yield (Supplementary Fig. S2). Nonetheless, addition of other *E*. *coli* PPIases (e.g., trigger factor^45,46^) or eukaryotic PPIases to the optimized PURE system may still further accelerate IgG formation and improve IgG formation efficiency. On the other hand, it has been reported that initial disulfide bond formation facilitates the establishment of a folding nucleus for the Ig domain^3^. Similarly, Sato *et al*. reported that PDI family proteins ERp46 and P5 act in concert with PDI to accelerate disulfide bond formation in the mammalian ER^47^. Those authors hypothesized that ERp46 and P5 rapidly and indiscriminately introduce non-native disulfide bonds to the nascent polypeptide chain while translocating the chain into the ER, and PDI proofreads the disulfide bond to ensure the formation of the correct links. Similarly, the prokaryotic DsbA protein has been predicted to introduce disulfide bonds acting in cooperation with DsbC^48,49^. Therefore, addition of P5 or DsbA to the optimized PURE system also may accelerate IgG formation and improve IgG formation efficiency.

In the present study, we used the optimized PURE system and a simple procedure to successfully synthesize trastuzumab and other IgG antibodies, including members of the IgG1, IgG2, and IgG4 subclasses, in the active form. The synthesis used PCR products as templates and was completed in a total of 2 days. The yield ranged from 33 ± 3 to 124 ± 9 μg/mL, which would be sufficient for small-scale evaluation experiments at the early stages of development of monoclonal antibodies, for example. The information obtained during optimization of the synthesis temperature and of the template DNA ratio for individual IgGs may provide important information for improving the stability of each IgG. On the other hand, in the case of synthesis of multiple IgGs at the same time with the optimized PURE system, a condition of 30 °C and *LC* DNA:*HC* DNA = 1:1 is recommended as the universal synthesis condition for high-throughput. The reaction mixture containing synthesized IgG was directly applied to ELISA and the affinities could be evaluated even without purification. Additionally, as we report in the supporting information, the level of harmful endotoxins (lipopolysaccharides) is low in the PURE*frex*^®^ kit, so the reaction mixture containing synthesized product could be directly applied to cell-based assays for functional screening when using more than 400-fold dilution without purification (Supplementary Fig. S6 and Supplementary Fig. S7). Although further work on topics such as disulfide bond isoform structure^50^, aggregation stability^51,52^, and IgG-Fc receptor binding^53^ is needed, our results demonstrated that the PURE system can be used as a platform for simple and robust high-throughput expression and functional screening for the development of monoclonal IgGs.

## Methods

### Preparation of template DNAs

All artificial genes encoding IgGs were designed from amino acid sequences registered in the public database (Drug Bank; https://www.drugbank.ca) and patent documents. To enhance protein productivity, AT-rich codons were selected at the N-terminal second to sixth codons of *LC* and *HC*, as described in several studies^54–56^; the ORF sequences also were optimized for *E*. *coli* codon usage. Each sequence was designed with a flanking 5′-UTR including the T7 promoter and the Shine-Dalgarno sequence (5′- GAAATTAATACGACTCACTATAGGGAGACCACAACGGTTTCCCTCTAGAAATAATTTTGTTTAACTTTAAGAAGGAGATATACCA-Start codon-ORF-3′) and a 3′-terminal adaptor sequence (5′-ORF-Stop codon-TGAATAACTAATCC-3′); these flanking sequences were attached to each ORF using PCR. The *HC* sequences of trastuzumab, adalimumab, and cetuximab were designed to encode a C-terminal FLAG-tag.

### Cloning, expression, and purification of chaperone-like proteins

DNAs encoding the mature region of FkpA (accession no. NP_417806, aa 26–270) and Skp (accession no. NP_414720, aa 21–161) were amplified from *E*. *coli* TG1 genomic DNA and cloned into the pET-15b expression vector. *E*. *coli* BL21 (DE3) cells were transformed with the vectors and N-terminal His-tagged recombinant proteins were expressed with 0.1 mM IPTG induction. The recombinant proteins were purified using Ni-affinity resins (Ni sepharose 6 Fast Flow, GE Healthcare, Little Chalfont, England) according to standard methods. Purified FkpA and Skp were concentrated and buffer-exchanged into 20 mM HEPES-KOH (pH 7.6), 200 mM potassium acetate, 1 mM DTT, and 10% glycerol.

### Cell-free protein synthesis

Cell-free protein synthesis was performed using PURE*frex 2*.*0* (GeneFrontier, Chiba, Japan) according to the manufacturer’s instructions. *LC* and *HC* template DNAs were added simultaneously to the reaction mixture as 10 nM mixed PCR products. DS supplement (7.5 mg/mL (320 μM) DsbC suspension and 60 mM GSSG solution; GeneFrontier) and DnaK mix (20× solution: 100 µM DnaK, 20 µM DnaJ, and 20 µM GrpE; GeneFrontier) were added at the indicated concentrations. For use as a reducing agent, GSH, TCEP, MPD, or 2-ME was added (to 2 mM) in place of DTT. Purified FkpA and Skp were added at 25 or 50 µM, depending on the specific experiment. Incubation was typically performed at 37 °C for 16 or 28 hours, except where indicated otherwise.

### Quantitative determination of cell-free synthesized proteins

All reaction mixtures containing synthesized products were centrifuged at 9,100 × g for 10 min, and then 0.5 µL and 1.0 µL of the supernatant was subjected to reducing (12.5% (w/v)) and non-reducing (10% (w/v)) SDS-PAGE, respectively. Serial dilutions of bovine serum albumin (BSA) protein standard (#23209, Thermo Fisher Scientific, Waltham, MA) also were applied to the SDS-PAGE as a quantity standard. The gels were stained with Oriole fluorescent gel stain (Bio-Rad, Hercules, CA). Protein bands were visualized and quantitated using the LAS-4000 system (GE Healthcare) and Multi Gauge ver. 3.1 software (FUJIFILM, Tokyo, Japan). The amounts of the total synthesized LC and HC (from reducing SDS-PAGE) and the whole IgG generated (from non-reducing SDS-PAGE) in the same sample were calculated from a standard curve of BSA. Unless otherwise noted, all quantitated data represent the average and standard deviations of three independent experiments. The IgG formation efficiency was defined as the proportion of LC and HC which formed IgG out of the total synthesized LC and HC molecules and was calculated by the following formula:

1 $$\mathrm{IgG}\,\mathrm{formation}\,\mathrm{efficiency}\,( \% )=\,4\,\times \,\frac{\mathrm{Whole}\,\mathrm{IgG}\,\mathrm{concentration}\,({\rm{\mu }}{\rm{M}})}{\mathrm{Total}\,\mathrm{LC}\,\mathrm{and}\,\mathrm{HC}\,\mathrm{concentration}\,({\rm{\mu }}{\rm{M}})}\,\times \,\mathrm{100}\,$$

### Purification of cell-free synthesized trastuzumab

One milliliter of the reaction mixture containing synthesized trastuzumab was added to 25 µL of Protein A cellulose resin (KANEKA KanCap A, KANEKA, Osaka, Japan) equilibrated with wash buffer (20 mM sodium phosphate buffer (pH 7.0), 0.05% Tween 20) and incubated with rotation for 1 hour at 4 °C. The resins were washed once with wash buffer supplemented with 20 mM MgCl_2_ and then three times with unsupplemented wash buffer. The bound trastuzumab was eluted with 50 mM glycine-HCl (pH 2.5), 0.05% Tween 20, and immediately adjusted to neutral pH with a small volume of 1 M Tris-HCl (pH 8.8). The eluate was applied to a gel filtration column (Superdex-200 10/300 GL, GE Healthcare) equilibrated with phosphate-buffered saline (PBS), 0.05% Tween 20 and fractionated at 0.5 mL/tube. Fractions including trastuzumab were concentrated by centrifugal filtration (Amicon Ultra-0.5 mL filters (30 K), Merck Millipore, Burlington, MA).

### Internalization analysis

The HER2-expressing cell line BT-474 was obtained from ATCC (Manassas, VA). Cells were maintained in Dulbecco’s Modified Eagle’s medium (DMEM) supplemented with 10% fetal bovine serum (FBS) and antibiotics at 37 °C in a 5% CO_2_ environment. Experiments were performed as described previously^32^. Cells (8000 cells/well) growing on poly-L-lysine-coated 96-well plates were surface-labeled with 10 nM purified IgG (obtained as described above) or authentic trastuzumab in binding medium (20 mM HEPES, 3% BSA, in serum-free DMEM, pH 7.4) at 4 °C for 1 hour. Cells were washed five times with binding medium and then incubated with binding medium at 37 °C for 0 or 3 hours. Cells then were fixed in 4% paraformaldehyde in PBS, and washed three times with 50 mM NH_4_Cl, 0.1 M glycine in PBS, before being permeabilized with saponin blocking buffer (0.4% saponin, 1% BSA, 2% normal goat serum in PBS) at 4 °C overnight. All subsequent primary and secondary antibody incubations and wash steps were performed in saponin blocking buffer. Trastuzumab was visualized with DyLight 549 AffiniPure Goat Anti-Human IgG, F(ab′)_2_ fragment-specific (1:500 dilution; #109-505-097, Jackson ImmunoResearch, West Grove, PA). CD63 was visualized with monoclonal anti-CD63 antibody produced in mouse (1:500 dilution; #SAB4700215, Sigma-Aldrich, St. Louis, MO) and Alexa Fluor-488-labeled goat anti-mouse IgG (1:500 dilution; #A11029, Invitrogen, Carlsbad, CA). Cells were imaged with an inverted fluorescence microscope system (Olympus, Tokyo, Japan).

### Binding kinetics analysis

Binding kinetics was measured by biolayer interferometry on an Octet RED96 system (Pall ForteBio, Fremont, CA). The purified IgG and authentic trastuzumab were diluted with Kinetics Buffer 10× (Pall ForteBio) and loaded onto Anti-Human IgG Fc Capture biosensor (Pall Fortebio) until 0.4 nm of binding was attained. The association (180 sec) and dissociation (300 sec) were measured with serial dilutions of recombinant HER2 (#10004-H08H, Sino Biological, Beijing, China) ranging from 17.6 to 1.1 nM. The dissociation constant (*K*_D_), association rate constant (*k*_on_) and dissociation rate constant (*k*_off_) were calculated using Octet Data Analysis 9.0 software (Pall ForteBio).

### Thermofluor assay

Thermofluor assay was performed as described previously^33^ using the ProteoStat Thermal Shift Stability Assay kit (Enzo Life Sciences, Farmingdale, NY). For each 25 µL reaction, 12.5 µL of the purified IgG or authentic trastuzumab (each 0.2 mg/mL solution in PBS), 10 µL of 1× Assay Buffer and 2.5 µL of 10 × ProteoStat TS Detection Reagent were mixed. Samples were heated from 20 to 99 °C at 5 °C/minute and fluorescence was read using the LightCycler 480 system (Roche, Basel, Switzerland) at 480 nm excitation and 610 nm emission. The fluorescent curves were plotted using LightCycler 480 software 1.5.1 (Roche).

### ELISA

Antigens for the respective synthesized IgGs were coated on 384-well plates at the following concentrations: 1.0 ng/well of Recombinant Human ErbB2/HER2 (#10004-H08H, Sino Biological), 40 ng/well of Recombinant Human TNF-alpha (#210-TA-020/CF, R&D Systems, Minneapolis, MN), 12.5 ng/well of Human EGFR/HER1/ErbB1 Protein (#10001-H08H-10, Sino Biological), and 20 ng/well of PD-1 (CD279) Fc Fusion (#71106, BPS Bioscience, San Diego, CA). The bound IgG was detected using anti-human Fab secondary antibody (1:1500 dilution in TBS-T; #109-035-097, Jackson ImmunoResearch).

## Electronic supplementary material

Supplementary information

## References

[1] Ecker DM, Jones SD, Levine HL (2015). The therapeutic monoclonal antibody market. MAbs..

[2] Schroeder HW, Cavacini L (2010). Structure and function of immunoglobulins. J Allergy Clin Immunol..

[3] Feige MJ, Hendershot LM, Buchner J (2010). How antibodies fold. Trends Biochem Sci..

[4] Kawasaki T, Gouda MD, Sawasaki T, Takai K, Endo Y (2003). Efficient synthesis of a disulfide-containing protein through a batch cell-free system from wheat germ. Eur J Biochem..

[5] Merk H, Gless C, Maertens B, Gerrits M, Stiege W (2012). Cell-free synthesis of functional and endotoxin-free antibody Fab fragments by translocation into microsomes. Biotechniques..

[6] Stech M, Hust M, Schulze C, Dübel S, Kubick S (2014). Cell-free eukaryotic systems for the production, engineering, and modification of scFv antibody fragments. Eng Life Sci..

[7] Merk H, Stiege W, Tsumoto K, Kumagai I, Erdmann VA (1999). Cell-free expression of two single-chain monoclonal antibodies against lysozyme: effect of domain arrangement on the expression. J Biochem..

[8] Jiang X, Ookubo Y, Fujii I, Nakano H, Yamane T (2002). Expression of Fab fragment of catalytic antibody 6D9 in an *Escherichia coli in vitro* coupled transcription/translation system. FEBS Lett..

[9] Ali M (2005). Improvements in the cell-free production of functional antibodies using cell extract from protease-deficient *Escherichia coli* mutant. J Biosci Bioeng..

[10] Galeffi P (2006). Functional expression of a single-chain antibody to ErbB-2 in plants and cell-free systems. J Transl Med..

[11] Yin G (2012). Aglycosylated antibodies and antibody fragments produced in a scalable *in vitro* transcription-translation system. MAbs..

[12] Frey S, Haslbeck M, Hainzl O, Buchner J (2008). Synthesis and characterization of a functional intact IgG in a prokaryotic cell-free expression system. Biol Chem..

[13] Groff D (2014). Engineering toward a bacterial “endoplasmic reticulum” for the rapid expression of immunoglobulin proteins. MAbs..

[14] Buntru M, Vogel S, Stoff K, Spiegel H, Schillberg S (2015). A versatile coupled cell-free transcription-translation system based on tobacco BY-2 cell lysates. Biotechnol Bioeng..

[15] Martin RW (2017). Development of a CHO-Based Cell-Free Platform for Synthesis of Active Monoclonal Antibodies. ACS Synth Biol..

[16] Stech M (2017). Cell-free synthesis of functional antibodies using a coupled *in vitro* transcription-translation system based on CHO cell lysates. Sci Rep..

[17] Shimizu Y (2001). Cell-free translation reconstituted with purified components. Nat Biotechnol..

[18] Niwa T (2009). Bimodal protein solubility distribution revealed by an aggregation analysis of the entire ensemble of *Escherichia coli* proteins. Proc Natl Acad Sci USA.

[19] Carter P (1992). Humanization of an anti-p185HER2 antibody for human cancer therapy. Proc Natl Acad Sci USA.

[20] Genevaux P, Georgopoulos C, Kelley WL (2007). The Hsp70 chaperone machines of *Escherichia coli*: a paradigm for the repartition of chaperone functions. Mol Microbiol..

[21] Castanié-Cornet MP, Bruel N, Genevaux P (2014). Chaperone networking facilitates protein targeting to the bacterial cytoplasmic membrane. Biochim Biophys Acta..

[22] Zapun A, Missiakas D, Raina S, Creighton TE (1995). Structural and functional characterization of DsbC, a protein involved in disulfide bond formation in *Escherichia coli*. Biochemistry..

[23] Fabianek RA, Hennecke H, Thöny-Meyer L (2000). Periplasmic protein thiol:disulfide oxidoreductases of *Escherichia coli*. FEMS Microbiol Rev..

[24] Goemans C, Denoncin K, Collet JF (2014). Folding mechanisms of periplasmic proteins. Biochim Biophys Acta..

[25] Levy R, Weiss R, Chen G, Iverson BL, Georgiou G (2001). Production of correctly folded Fab antibody fragment in the cytoplasm of *Escherichia coli* trxB gor mutants via the coexpression of molecular chaperones. Protein Expr Purif..

[26] Zhang Z (2003). Production of soluble and functional engineered antibodies in *Escherichia coli* improved by FkpA. Biotechniques..

[27] Entzminger KC, Chang C, Myhre RO, McCallum KC, Maynard JA (2012). The Skp chaperone helps fold soluble proteins *in vitro* by inhibiting aggregation. Biochemistry..

[28] Levy R (2013). Enhancement of antibody fragment secretion into the *Escherichia coli* periplasm by co-expression with the peptidyl prolyl isomerase, FkpA, in the cytoplasm. J Immunol Methods..

[29] Wang R (2013). Engineering production of functional scFv antibody in *E*. *coli* by co-expressing the molecule chaperone Skp. Front Cell Infect Microbiol..

[30] Ramm K, Plückthun A (2000). The periplasmic *Escherichia coli* peptidylprolyl cis,trans-isomerase FkpA. II. Isomerase-independent chaperone activity *in vitro*. J Biol Chem..

[31] Matsubayashi H, Kuruma Y, Ueda T (2014). *In vitro* synthesis of the *E*. *coli* Sec translocon from DNA. Angew Chem Int Ed Engl..

[32] Austin CD (2004). Endocytosis and sorting of ErbB2 and the site of action of cancer therapeutics trastuzumab and geldanamycin. Mol Biol Cell..

[33] McConnell AD (2014). A general approach to antibody thermostabilization. MAbs..

[34] Mimura Y (2001). Role of oligosaccharide residues of IgG1-Fc in Fc gamma RIIb binding. J Biol Chem..

[35] Zheng K, Bantog C, Bayer R (2011). The impact of glycosylation on monoclonal antibody conformation and stability. MAbs..

[36] Bonomo J, Welsh JP, Manthiram K, Swartz JR (2010). Comparing the functional properties of the Hsp70 chaperones, DnaK and BiP. Biophys Chem..

[37] Chen J (1999). Chaperone activity of DsbC. J Biol Chem..

[38] Chau MH, Nelson JW (1991). Direct measurement of the equilibrium between glutathione and dithiothreitol by high performance liquid chromatography. FEBS Lett..

[39] Rothwarf DM, Scheraga HA (1992). Equilibrium and kinetic constants for the thiol-disulfide interchange reaction between glutathione and dithiothreitol. Proc Natl Acad Sci USA.

[40] Szajewski RP, Whitesides GM (1980). Rate constants and equilibrium constants for thiol-disulfide interchange reactions involving oxidized glutathione. J. Am. Chem. Soc..

[41] Burns JA, Butler JC, Moran J, Whitesides GM (1991). Selective reduction of disulfides by tris(2-carboxyethyl)phosphine. J. Org. Chem..

[42] Lilie H, Lang K, Rudolph R, Buchner J (1993). Prolyl isomerases catalyze antibody folding *in vitro*. Protein Sci..

[43] Jäger M, Plückthun A (1997). The rate-limiting steps for the folding of an antibody scFv fragment. FEBS Lett..

[44] Feige MJ (2009). An unfolded CH1 domain controls the assembly and secretion of IgG antibodies. Mol Cell..

[45] Hesterkamp T, Bukau B (1996). The *Escherichia coli* trigger factor. FEBS Lett..

[46] Hoffmann, A., Bukau, B. & Kramer, G. Structure and function of the molecular chaperone Trigger Factor. *Biochim Biophys Acta.* **1803**(6), 650–661 (2010). 10.1016/j.bbamcr.2010.01.017 20132842

[47] Sato Y (2013). Synergistic cooperation of PDI family members in peroxiredoxin 4-driven oxidative protein folding. Sci Rep..

[48] Rietsch A, Belin D, Martin N, Beckwith J (1996). An *in vivo* pathway for disulfide bond isomerization in *Escherichia coli*. Proc Natl Acad Sci USA.

[49] Sone M, Akiyama Y, Ito K (1997). Differential *in vivo* roles played by DsbA and DsbC in the formation of protein disulfide bonds. J Biol Chem..

[50] Liu H, May K (2012). Disulfide bond structures of IgG molecules: structural variations, chemical modifications and possible impacts to stability and biological function. MAbs..

[51] Rouet R, Lowe D, Christ D (2014). Stability engineering of the human antibody repertoire. FEBS Lett..

[52] Geng SB, Cheung JK, Narasimhan C, Shameem M, Tessier PM (2014). Improving monoclonal antibody selection and engineering using measurements of colloidal protein interactions. J Pharm Sci..

[53] Vidarsson G, Dekkers G, Rispens T (2014). IgG subclasses and allotypes: from structure to effector functions. Front Immunol..

[54] Allert M, Cox JC, Hellinga HW (2010). Multifactorial determinants of protein expression in prokaryotic open reading frames. J Mol Biol..

[55] Bentele K, Saffert P, Rauscher R, Ignatova Z, Blüthgen N (2013). Efficient translation initiation dictates codon usage at gene start. Mol Syst Biol..

[56] Boël G (2016). Codon influence on protein expression in *E*. *coli* correlates with mRNA levels. Nature..

