# Supplementary material for: Constructive approach for synthesis of a functional IgG using a reconstituted cell-free protein synthesis system
Source: Sci Rep. 2019 Jan 24;9:671. doi: 10.1038/s41598-018-36691-8 (PMC6345822; doi:10.1038/s41598-018-36691-8)
Supplement: Supplementary file 1 — Supplementary information [file 41598_2018_36691_MOESM1_ESM.pdf]

Supplementary Information

Constructive approach for synthesis of a functional IgG using a reconstituted cell-free protein synthesis system

Satoshi Murakami*, Rena Matsumoto & Takashi Kanamori*

GeneFrontier Corporation

*Addresses correspondence to: Satoshi Murakami and Takashi Kanamori,
Todai-Kashiwa Venture Plaza #308, 5-4-19 Kashiwanoha, Kashiwa, Chiba 277-0882, Japan.
Tel: +81-4-7137-6301; Fax: +81-4-7132-7530
E-Mail: murakami@genefrontier.com, kanamori@genefrontier.com

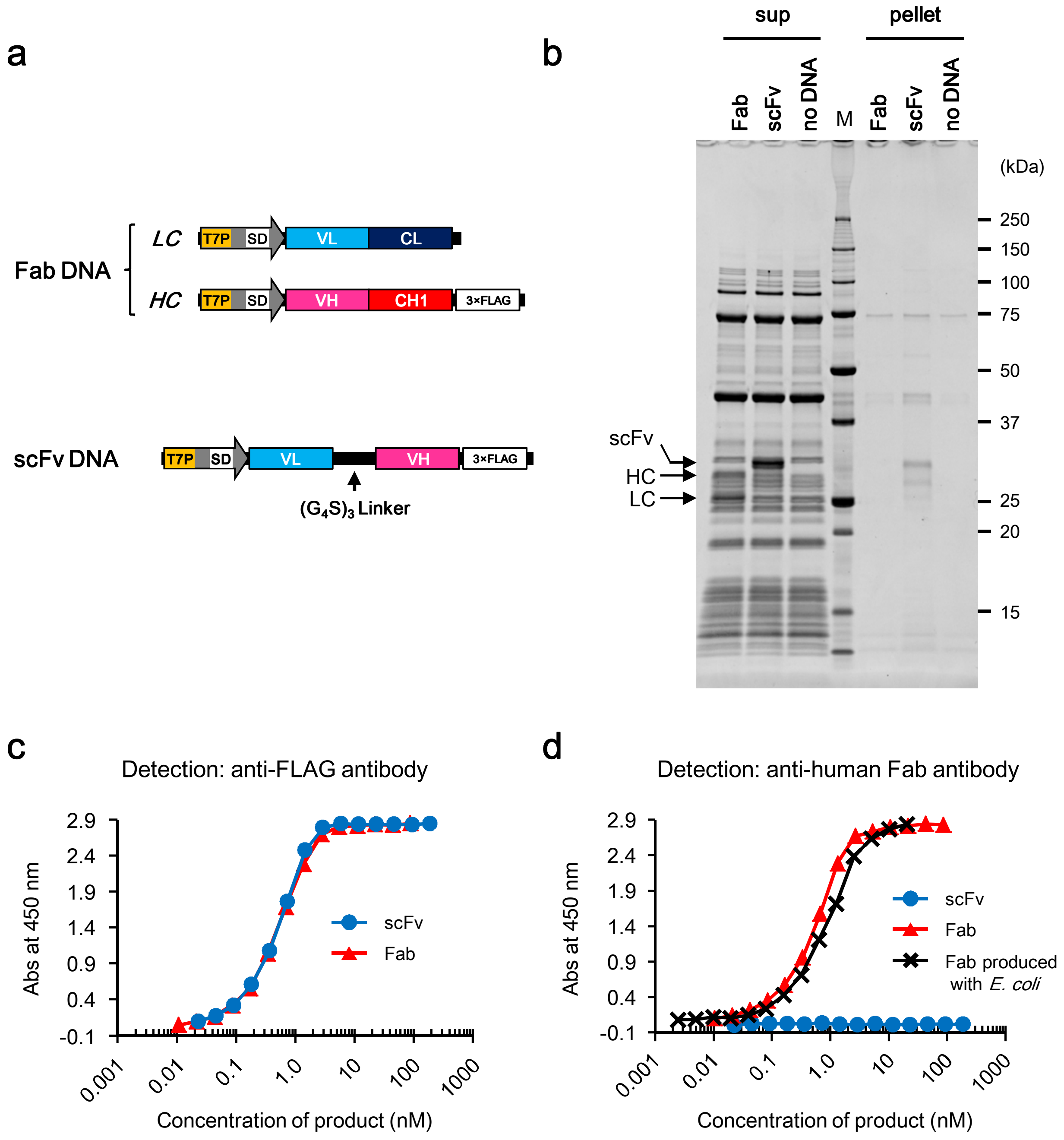

Supplementary Fig. S1. Synthesis of Fab and scFv derived from trastuzumab using the PURE system. (**a**) Design of template DNAs encoding Fab and scFv. In the case of scFv, the VL and VH domains were connected via three tandem glycine-serine (G_4S) linkers. T7P: T7 promoter; SD: Shine-Dalgarno sequence. (**b**) Synthesis of Fab and scFv. The synthesis reaction was performed with the PURE system containing 3 mM GSSG, 1 μM DsbC, and 1× DnaK mix. The mixed template DNAs encoding Fab (molar ratio of *LC*:*HC*=1:4) or encoding scFv were added to the reaction mixture to a total concentration of 10 nM. Incubation was performed at 37°C for 16 hours. After synthesis, the reaction mixture was centrifuged and separated into supernatant (sup) and pellet and subjected to reducing SDS-PAGE (10-20% gradient gel). The SDS-PAGE gel was stained with fluorescent dye. M, molecular weight marker. (**c** and **d**) Analysis of the binding of synthesized Fab and scFv to the antigen. The reaction mixture containing the synthesized product was serially diluted and subjected to ELISA. Binding was detected with (**c**) anti-FLAG M2-HRP antibody (1:2000 dilution in TBS-T; #A8592, Sigma-Aldrich) or (**d**) anti-human Fab secondary antibody (1:2000 dilution in TBS-T; #109-035-097, Jackson ImmunoResearch). Fab synthesized using the PURE system showed the same activity as purified Fab produced in *E. coli*.

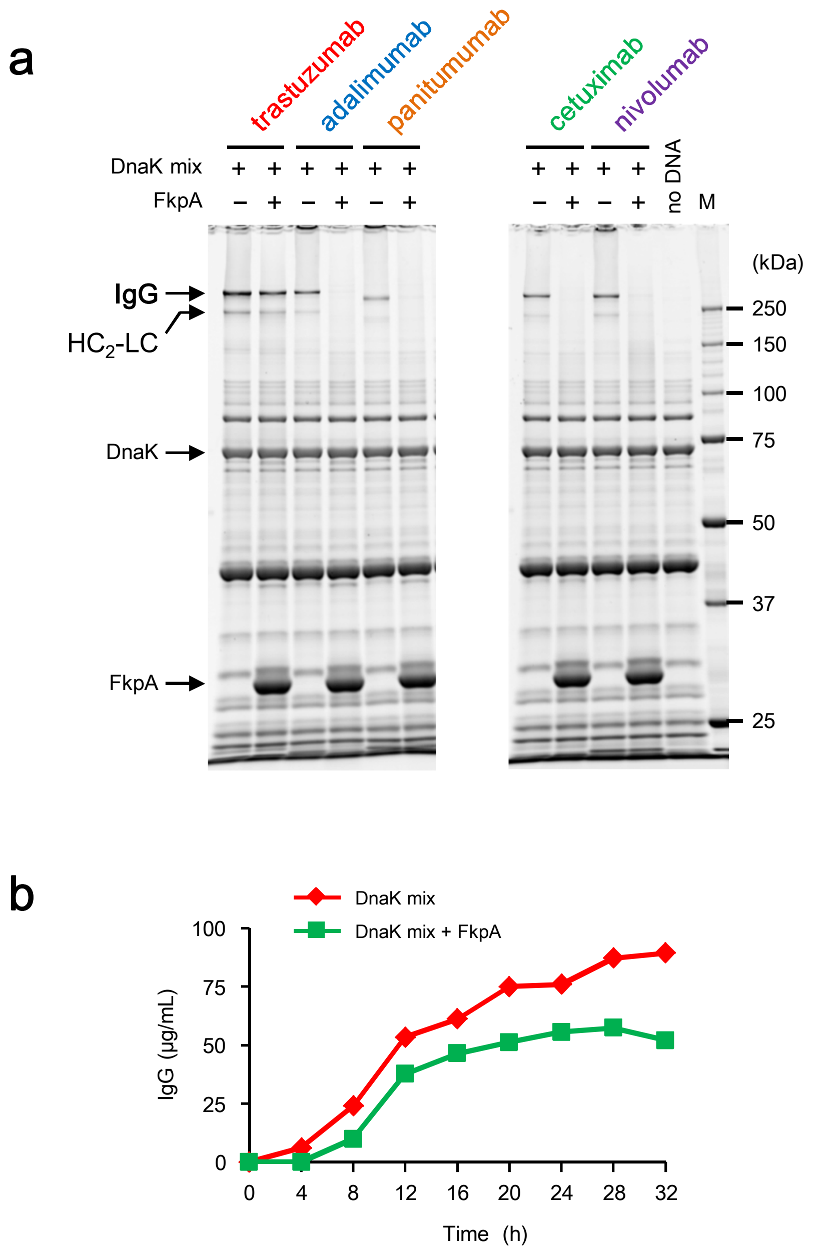

Supplementary Fig. S2. Investigation of additive effect of DnaK mix and FkpA on IgG formation. (**a**) Synthesis of several IgGs in the presence of 1× DnaK mix with or without FkpA. The reaction was carried out with the optimized reaction mixture shown in Fig. 5a with or without addition of 25 µM FkpA. Incubation was performed for 28 hours. Synthesis temperature and template DNA ratio (molar ratio of *LC*:*HC*) were optimized for the individual IgGs as shown in Table 1. After centrifugation, the supernatant was subjected to non-reducing SDS-PAGE (10% gel). Images (trastuzumab, adalimumab and panitumumab; cetuximab and nivolumab) were obtained from separate gels. M, molecular weight marker. (**b**) Time course of IgG formation of trastuzumab in the presence of 1× DnaK mix with or without 25 µM FkpA. The amount of whole IgG was quantitated from non-reducing SDS-PAGE. The values represent the means of duplicate experiments.

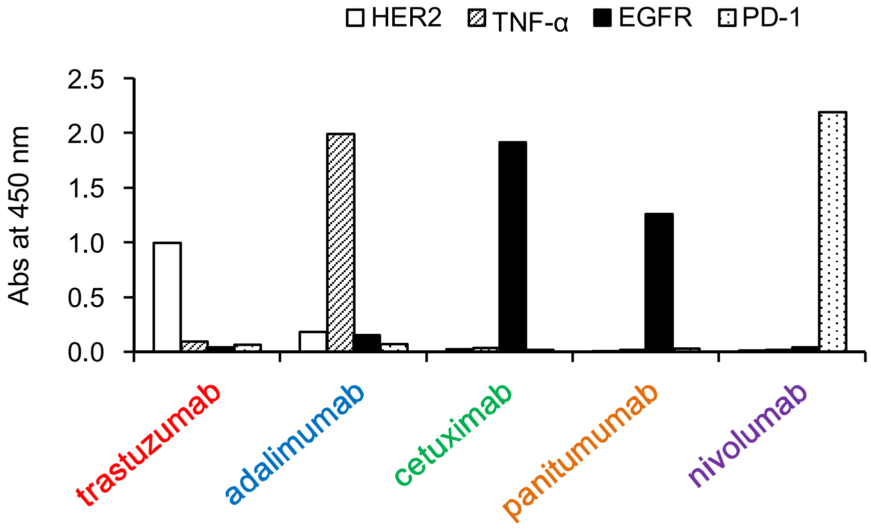

Supplementary Fig. S3. Check of cross-reactivity of synthesized IgGs by ELISA. The reaction mixture containing synthesized IgG was diluted 200-fold with TBS-T and applied to ELISA under the same conditions as in Fig. 7b.

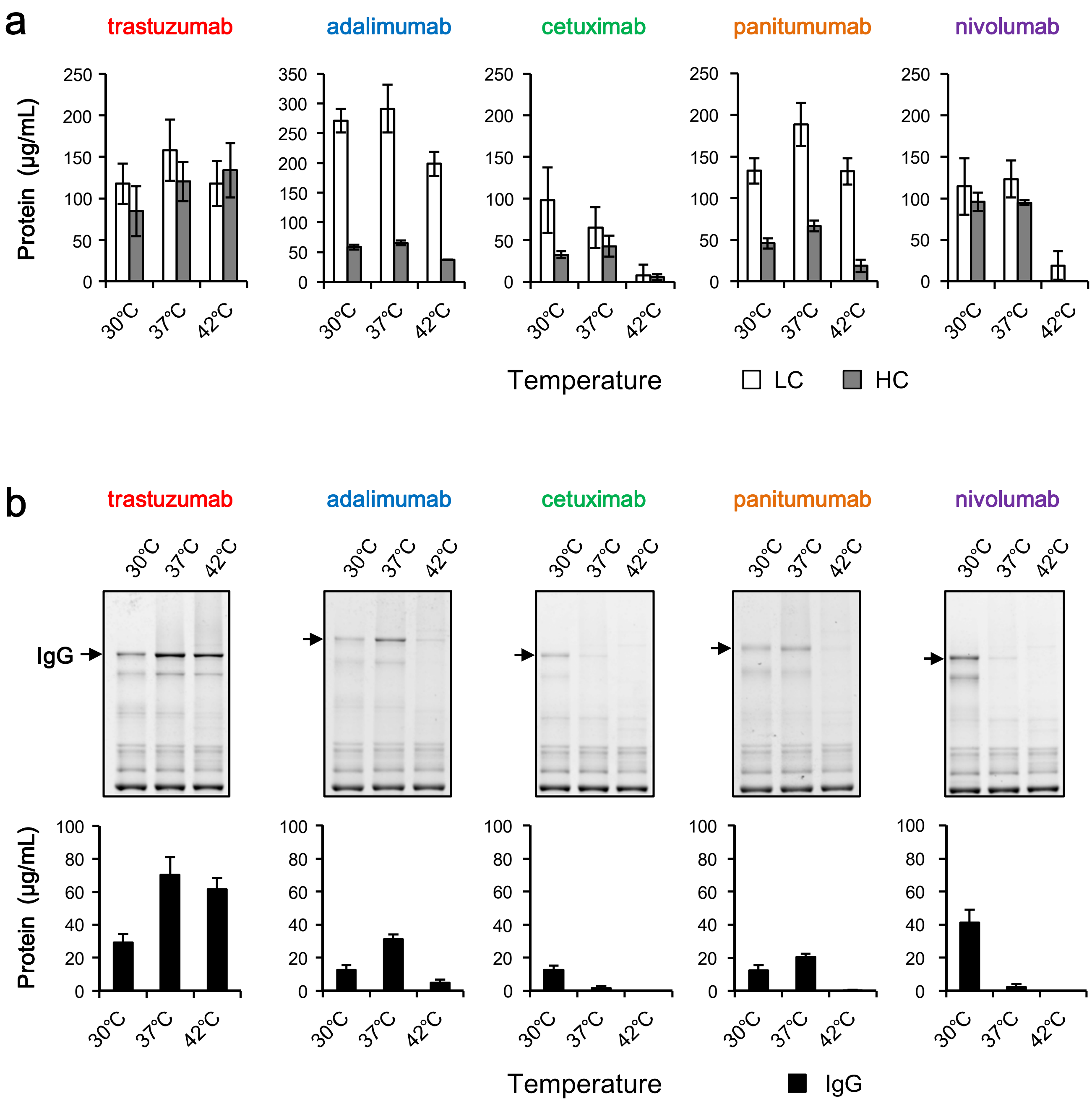

Supplementary Fig. S4. Optimization of synthesis temperature of several IgGs. The reactions were performed with the PURE system containing 2 mM GSH instead of DTT, 3 mM GSSG, 5 µM DsbC, 1× DnaK mix, and 10 nM mixed template DNA (molar ratio of *LC*:*HC*=1:1). Incubations were performed at 30, 37, and 42°C for 16 hours. After centrifugation, the supernatant fractions were subjected to quantitative analysis. The total soluble LC and HC (**a**) and the whole IgG (**b**) in the same sample were quantitated with reducing and non-reducing SDS-PAGE with different gels (12.5% and 10% gel, respectively). All data represent the mean and standard deviation of three independent experiments.

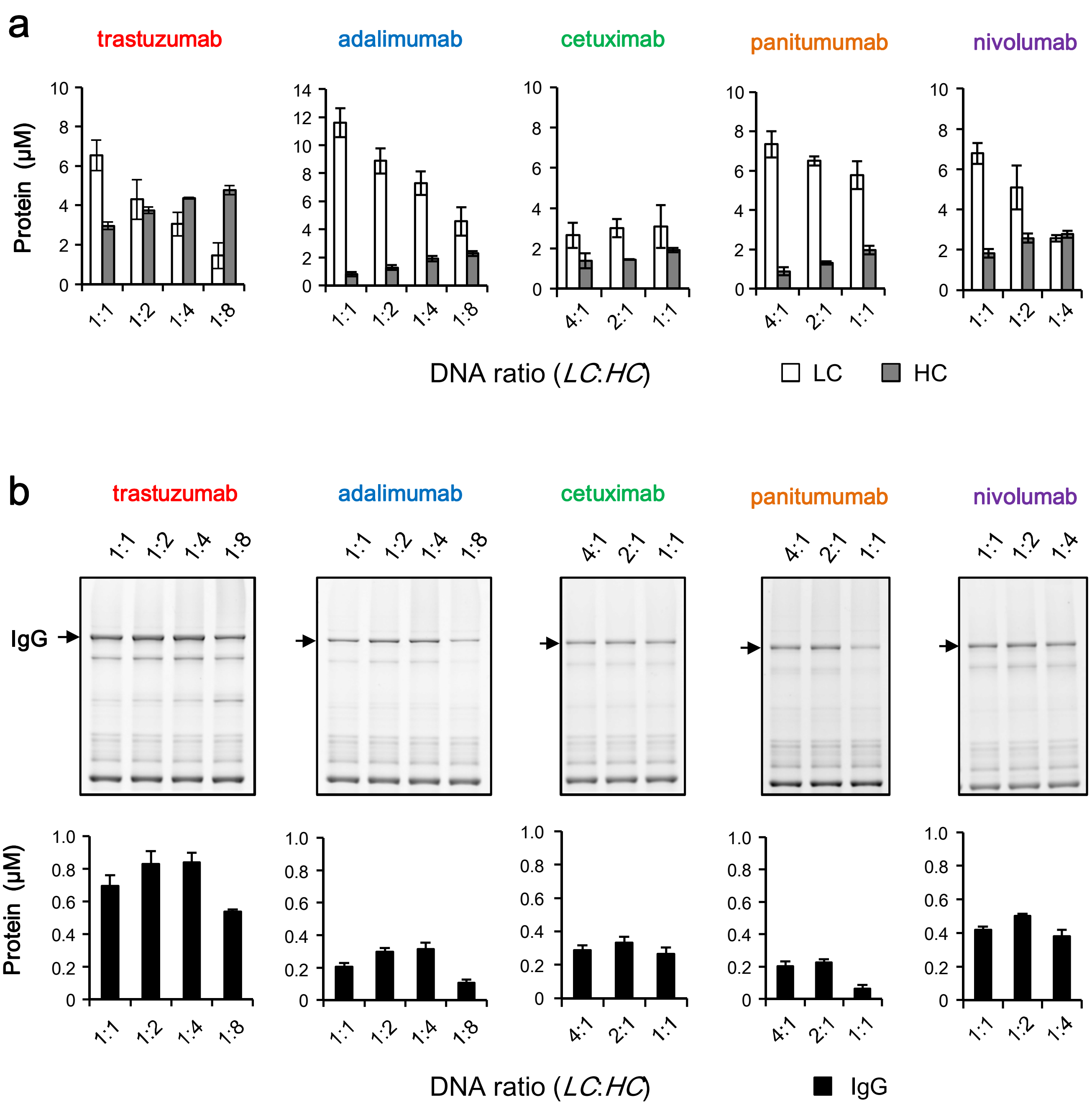

Supplementary Fig S5. Optimization of template DNA ratio for several IgGs. The reactions were performed with the PURE system containing 2 mM GSH instead of DTT, 3 mM GSSG, 5 μM DsbC, 1× DnaK mix, and 10 nM mixed template DNA (at various molar ratios of *LC* DNA and *HC* DNA). Incubations were performed for 28 hours at each optimal temperature as shown in Table 1. After centrifugation, the supernatant fractions were subjected to quantitative analysis. The total soluble LC and HC (**a**) and the whole IgG (**b**) in the same sample were quantitated with reducing and non-reducing SDS-PAGE with different gels (12.5% and 10% gel, respectively). All data represent the mean and standard deviation of three independent experiments.

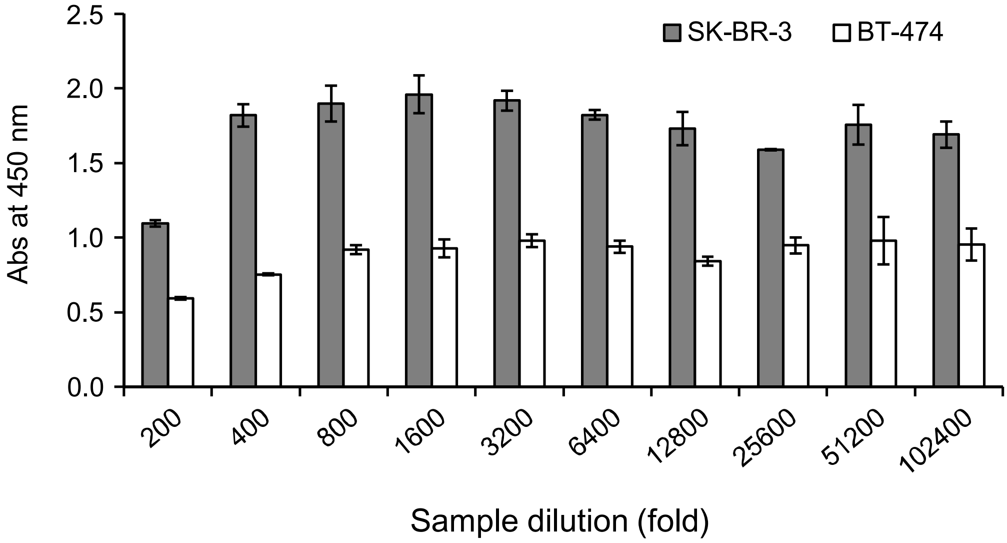

Supplementary Fig S6. The effect of contaminating endotoxin in the PURE*frex* kit on culture cell viability. Breast cancer cell lines SK-BR-3 and BT-474 were cultured with the medium (DMEM, 10% FBS, penicillin/streptomycin) containing serial dilutions of PURE*frex 2.0* reaction mixture without template DNA at 37°C for 4 days (SK-BR-3) or 6 days (BT-474). Cell viability was determined by the WST-8 method (Cell Counting kit-8, Dojindo, Tokyo, Japan); reactions were read at an absorbance of 450 nm. The values represent the mean and standard deviation of three independent experiments.

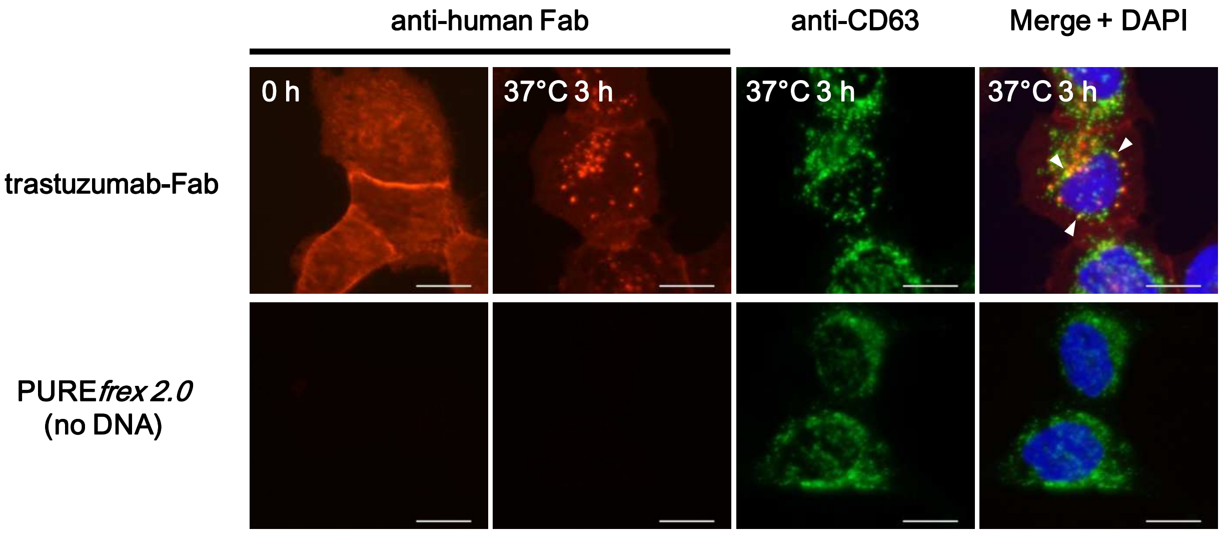

Supplementary Fig. S7. Direct addition of PURE*frex 2.0* reaction mixture to the cellular internalization analysis. The reaction mixture containing the synthesized Fab derived from trastuzumab (shown in Supplementary Fig. S1) was diluted 400-fold with the binding medium (20 mM HEPES, 3% BSA, in serum-free DMEM, pH 7.4). The reaction mixture without template DNA also was diluted and used in the assay as a negative control. BT-474 cells were surface-labeled at 4°C for 60 min with the diluted reaction mixture. Cells were washed and incubated at 37°C for 0 or 3 hours, and then fixed and processed by dual-label indirect immunofluorescence microscopy. CD63 (Lamp3) is a marker of late endosomes and lysosomes. White arrowheads indicate partial co-localization of the Fab with CD63. Bar indicates 20 μm.
